# Laparoscopic sleeve gastrectomy in a patient with primary adrenal insufficiency: a case report

**DOI:** 10.1093/jscr/rjaf156

**Published:** 2025-04-02

**Authors:** Abdulrhman Ahmed Nemri, Khalid Alshehri, Amer Alshahrani, Monera Abdulhaq, Abrar Hamad Alageel

**Affiliations:** Security Forces Hospital in Riyadh, General Surgery, Salah Ad Din Al Ayyubi Rd, Riyadh 11481, Saudi Arabia; Security Forces Hospital in Riyadh, Surgery, Salah Ad Din Al Ayyubi Rd, Riyadh 11564, Saudi Arabia; Security Forces Hospital in Riyadh, General Surgery, Salah Ad Din Al Ayyubi Rd, Riyadh 11481, Saudi Arabia; Prince Sultan Military Medical City, Internal Medicine, Makkah Al Mukarramah Rd, Riyadh 11159, Saudi Arabia; Saudi Arabia Ministry of Health, General Surgery, Riyadh 11176, Saudi Arabia

**Keywords:** bariatric surgery, morbid obesity, adrenal insufficiency, sleeve gastrectomy

## Abstract

This case report discusses the successful application of laparoscopic sleeve gastrectomy (LSG) in a 17-year-old patient diagnosed with primary adrenal insufficiency (PAI). The patient presented with severe obesity (BMI > 60 kg/m^2^) along with metabolic issues, including dyslipidemia and pre-diabetes. After failing conservative weight loss approaches and with significant binge eating behaviors, surgical intervention was deemed necessary. Despite the risks associated with steroid use in the surgical context, the patient underwent LSG with careful perioperative steroid management. His post operative recovery was uneventful, with no complications or adrenal crisis. Over the course of one year, he achieved a weight loss of 69 kg, reflecting a 37.7% reduction, indicating a significant improvement in his health status. This report highlights the feasibility and safety of LSG in patients with PAI and provides valuable insights into bariatric surgery outcomes in this unique population.

## Introduction

Bariatric surgery is widely recognized as the most effective treatment for severe obesity [1, 2], significantly reducing the associated morbidity and mortality while enhancing the overall quality of life [1, 3]. Bariatric surgery is considered an excellent option in reducing weight and improving quality of life compared to conservative management especially in sever obesity [4]. Although numerous studies have documented the efficacy of bariatric surgery in patients with secondary adrenal insufficiency and hypothalamic disorders [1, 5, 6], this is the first report of a patient with primary adrenal insufficiency (PAI) who underwent laparoscopic sleeve gastrectomy (LSG).

## Case report

A 17-year-old male with a medical history of dyslipidemia, pre-diabetes, and morbid obesity (BMI > 60 kg/m^2^) presented to his family physician with complaints of chronic fatigue, syncope, and recurrent hypoglycemic episodes. Laboratory investigations revealed a low cortisol level (38 ng/ml; normal range 171–536 ng/ml) and an adrenocorticotropic hormone (ACTH) level of 1.5 pg/ml (reference range 7.2–63.3 pg/ml). A Synacthen test and brain MRI confirmed the diagnosis of PAI, leading to the initiation of steroid replacement therapy.

To address his morbid obesity, the patient initially attempted weight reduction injections but experienced intolerable abdominal colic and dizziness, prompting discontinuation. During follow-up with bariatric surgery, a detailed assessment revealed binge eating patterns characterized by the consumption of large quantities of food for pleasure, coupled with a sedentary lifestyle. He reported consuming five to six cans of soft drinks daily and denied any symptoms of gastroesophageal reflux disease, although he exhibited signs suggestive of obstructive sleep apnea. His family history was notable for morbid obesity and several relatives had undergone bariatric surgery.

On examination, the patient was 173 cm tall, weighed 183 kg, and had a BMI of 61. Given the lack of improvement in lifestyle modifications, surgical intervention was deemed necessary. The patient was evaluated by psychiatry, nutrition, pulmonology, and anesthesia and ultimately received clearance for general anesthesia despite high-risk factors. Esophagogastroduodenoscopy and gallbladder ultrasonography yielded unremarkable results.

The patient underwent LSG with perioperative stress and a tapered steroid dose. His postoperative recovery was uneventful, with no signs of adrenal crisis, bleeding, or cardiopulmonary complications, resulting in discharged on the second postoperative day.

Regular follow-up appointments at 1 week, 1 month, 3 months, 6 months, and 1 year post operatively indicated excellent adherence to dietary recommendations. The patient lost ~30 kg in the first month, experienced proper wound healing, and successfully transitioned to a regular diet. At the 1-year follow-up, his weight had decreased to 114 kg, representing a 37.7% reduction.

## Discussion

Adrenal insufficiency (AI) is a potentially life-threatening yet treatable condition. PAI and Addison’s disease are rare causes of AI [7, 8]. Although there are multiple publications on patients with central adrenal insufficiency who have undergone different bariatric surgeries [1, 5, 6, 9], to our knowledge, this is the first reported case of laparoscopic sleeve gastrectomy in a patient with PAI.

Chronic steroid use increases the risk of post operative complications. Kaplan *et al.* [10] showed that patients on steroids undergoing bariatric surgery had a higher 30-day mortality rate of 3.4-folds, and double the 30-day readmission rate. Another retrospective study comparing chronic steroid users and non-users showed that the re-admission rate was significantly higher in steroid users compared to non-users (16.1% and 5.2%, respectively). Leaks and infections constituted 50% of readmission cases [11]. Mazzei *et al.* [12] collected data for almost 5000 steroid users who underwent LSG or Roux-en-Y gastric bypass (RYGB) using the Metabolic and Bariatric Surgery Accreditation Quality Improvement Program database. Steroid users had significantly higher complications, however, those patients were older and with significantly higher comorbidities, and with a matched analysis, steroid use was not found to be an independent predictor for worse outcomes; and deemed bariatric surgery a safe option for steroid users.

The outcomes of bariatric surgery in patients with AI vary among studies. A 12-month follow-up after LSG and RYGB showed similar weight reductions of 19.6% and 20.2%, respectively [5]. Nevertheless, another retrospective matched case–control study with a 2-year follow-up showed that LSG had a clinically significant lower weight loss reduction of only 12% at 1-year and 10% at 2-years, compared with 25% at 1-year and 2-year for RYGB patients [6]. In our case, the patient lost 69 kg at 1-year post operatively, constituting a 37.7% decrease in weight.

To our knowledge, this is the first report of LSG in a patient with PAI. Although various studies have shown less weight reduction in secondary AI, our patient had a weight loss comparable to that of the general population and non-steroid users. While steroid use does increase post operative morbidities in young and otherwise healthy patients, bariatric surgery remains a safe option in patients with PAI.
